# PARP inhibitors in ovarian cancer: Clinical evidence for informed treatment decisions

**DOI:** 10.1038/bjc.2015.395

**Published:** 2015-12-15

**Authors:** Jonathan A Ledermann, Fatima El-Khouly

**Affiliations:** 1 UCL Cancer Institute, University College London and UCL Hospitals Biomedical Research Centre, 90 Tottenham Court Road, London W1T 4TJ, UK

**Keywords:** olaparib, ovarian cancer, *BRCA*, PARP inhibitor, PFS, TFST, TSST

## Abstract

Ovarian cancer is the fifth leading cause of female cancer deaths in the Western world. Significant progress has been made in the treatment of patients with ovarian cancer, however, the majority of patients experience disease recurrence and new therapies are being sought for such patients. Clinical investigation of poly(ADP-ribose) polymerase (PARP) inhibitors for ovarian cancer treatment has demonstrated promising activity in this disease. Here, we review the development of PARP inhibitors and their future role in the treatment of patients with ovarian cancer. Studies of olaparib, the first PARP inhibitor to be approved in Europe and the USA, in patients with recurrent ovarian cancer have demonstrated clinical efficacy with improvements in progression-free survival. In maintenance therapy of platinum-sensitive ovarian cancer there is supporting evidence of clinical benefit from exploratory endpoints that include time to first subsequent treatment and time to second subsequent treatment. Adverse events that should be monitored following treatment with PARP inhibitors include nausea, vomiting, fatigue and anaemia. Based on the evidence presented, patients who will receive the greatest benefit from PARP inhibition are those with platinum-sensitive relapsed ovarian cancer and a *BRCA* mutation.

In developed countries, ovarian cancer is the fifth leading cause of female cancer deaths (American Cancer Society, 2013; Ferlay *et al*, 2013). Five-year survival rates for patients with ovarian cancer are 44% across all disease stages and 27% for advanced disease (Siegel *et al*, 2014). A large proportion of women with advanced disease will enter a remission following surgery and chemotherapy, but about 80% will relapse (National Cancer Institute, 2014). For those relapsing after an interval of more than 6 months, retreatment with platinum-based chemotherapy leads to a tumour response in the majority of women and control of the disease for a median of about 10–12 months (Fung-Kee**-**Fung *et al*, 2007; Colombo *et al*, 2010; Hanker *et al*, 2012; Ledermann *et al*, 2013). Tumours in this group of women are termed ‘platinum sensitive’ because of the high probability of response to platinum-based drugs (Friedlander *et al*, 2011). There is a clear need to improve outcome in these women, extending the time the disease remains under control, delaying the need for further therapy whilst maintaining their health-related quality of life, and ultimately developing therapies that improve survival.

One strategy is to use novel targeted maintenance therapies given after chemotherapy to maintain disease control. The advantage of this approach is that targeted treatments are more likely to be effective if the residual tumour burden is small. Maintenance drugs should be taken for a protracted period to sustain disease control; therefore they should be well tolerated, with limited side effects and little detrimental effect on quality of life.

Here, we discuss the strategy of using the first approved poly(ADP-ribose) polymerase (PARP) inhibitor olaparib (Lynparza), as maintenance treatment following platinum-based chemotherapy for relapsed ovarian cancer.

## For which patients are PARP inhibitors intended?

PARP is a key enzyme for the repair of single-strand DNA breaks via the base excision pathway. Inhibition of PARP leads to an accumulation of double-strand DNA breaks, resulting in the activation of homologous recombination repair (Farmer *et al*, 2005; Ashworth, 2008). However, homologous recombination deficiency (HRD) can arise from defects in the *BRCA* gene and also through BRCA-independent mechanisms (McCabe *et al*, 2006). HRD is particularly common in high-grade serous ovarian tumours, reported as up to 50% (Cancer Genome Atlas Research Network, 2011) and is a major feature of platinum-sensitive disease. In cancers with HRD, PARP inhibition leads to the formation of double-strand DNA breaks that cannot be repaired (Farmer *et al*, 2005), a concept known as synthetic lethality (Ashworth, 2008).

Preclinical models showed that inhibition of PARP-1 activity in models deficient in homologous recombination repair could lead to a potentially wide therapeutic index (Bryant *et al*, 2005; Farmer *et al*, 2005). Targeting of PARP inhibitors (PARPi) to *BRCA*-mutated (*BRCA*m) tumour cells over normal (non-tumour) cells in patients with *BRCA*m ovarian cancer should result in a favourable response. PARPi could, for the first time, offer personalised targeted therapy for patients with *BRCA1-* and/or *BRCA2*-mutated ovarian cancer. The scope of activity of PARPi is likely to be increased as measurement of HRD becomes more reliable.

## The pivotal Phase II olaparib study

The most studied PARPi to date is olaparib, with a number of completed and published Phase I and II clinical trials in ovarian cancer. The earliest studies were confined to patients with *BRCA* mutations, but PARPi have been clearly shown to be active in ovarian cancer without a *BRCA* mutation (Fong *et al*, 2009; Fong *et al*, 2010; Audeh *et al*, 2010; Gelmon *et al*, 2011; Kaye *et al*, 2012; Ledermann *et al*, 2012, 2014b; Oza *et al*, 2015). Studies have compared olaparib to pegylated liposomal doxorubicin in patients with *BRCA*m tumours (Kaye *et al*, 2012) and during and after combination with carboplatin and paclitaxel in platinum-sensitive recurrent ovarian cancer (Oza *et al*, 2015). The greatest activity appears to be in patients with platinum-sensitive tumours, although good tumour response rates and duration of response are seen in platinum-resistant *BRCA*m ovarian cancer (Kaye *et al*, 2012; Kaufman *et al*, 2015).

The investigation of olaparib maintenance therapy in platinum-sensitive recurrent ovarian cancer became the key strategy that led to the pivotal trial and subsequently several Phase III studies with different PARPi. The Phase II olaparib study (Study 19) was an international, multicentre, randomised, double-blind, placebo-controlled trial of olaparib maintenance treatment in patients with ovarian cancer who had responded to platinum-based chemotherapy (ClinicalTrials.gov NCT00753546; Ledermann *et al*, 2012). In this study, 265 patients were randomised to olaparib or placebo, and the primary end point was progression-free survival (PFS) by response evaluation criteria in solid tumours. Key inclusion criteria were that patients should have completed at least two previous courses of platinum-based chemotherapy regimens before randomisation, in response to their last platinum treatment, and were considered to have platinum-sensitive disease.

The Study 19 trial demonstrated a statistically significant improvement in PFS in patients receiving olaparib compared with placebo. In patients receiving olaparib, there was a 3.6-month increase in the median PFS from the start of trial drug (median PFS of 4.8 months *vs* 8.4 months for patients treated with olaparib and placebo, respectively; hazard ratio (HR) 0.35; 95% confidence interval (CI) 0.25–0.49; *P*<0.0001; Ledermann *et al*, 2012).

In addition, a retrospective, preplanned analysis of data by *BRCA* mutation status (germline or somatic) was also conducted. Blood and archival tumour samples provided information on *BRCA* mutation status in 95.8% of patients. Analyses of PFS and overall survival (OS) were performed for the overall population and by *BRCA* mutation status, as well as two exploratory clinical endpoints (time to first subsequent therapy or death (TFST; a clinically relevant interpretation of PFS, representing the clinical decision made by investigators to initiate a further course of chemotherapy); time to second subsequent therapy or death (TSST)), an approximation to the PFS2 (time to progression after subsequent treatment; Ledermann *et al*, 2014b).

**Figure 1:**
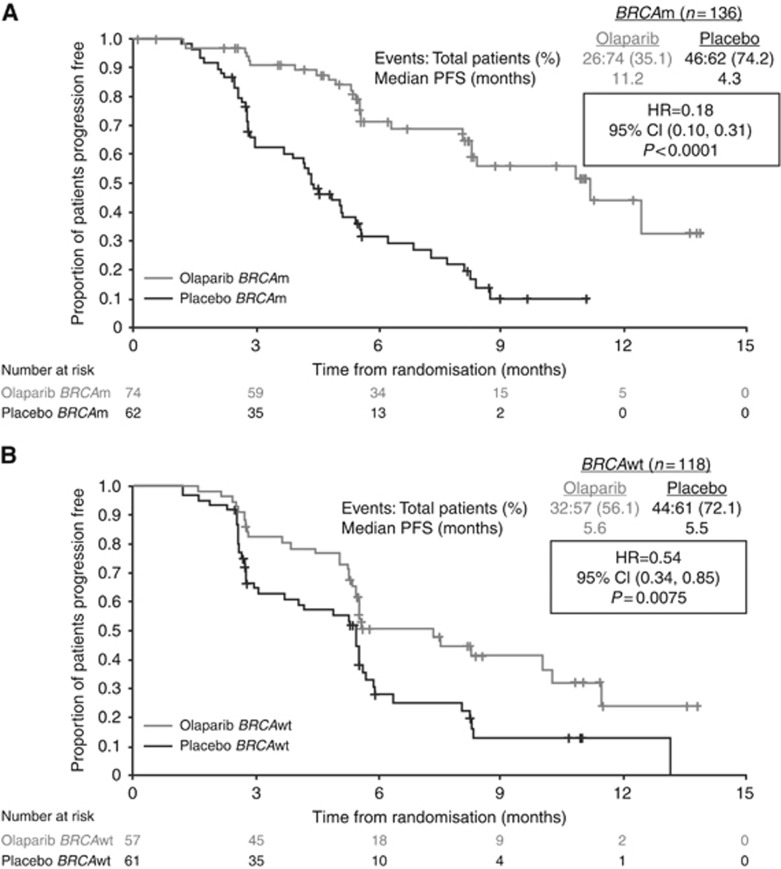
Progression-free survival in (**A**) patients with a *BRCA* mutation and (**B**) in patients with wild-type *BRCA* from the pivotal Phase II olaparib maintenance study in patients with platinum-sensitive relapsed serous ovarian cancer (Ledermann *et al*, 2012, 2014b). BRCAwt, wild type (includes patients with no known *BRCA* mutation or a mutation of unknown significance). Reprinted from Ledermann J *et al* (© 2014 with permission from Elsevier).

The clinical benefit of olaparib was greatest in the *BRCA*m group. In this subset of 136 patients, the median PFS postchemotherapy was 11.2 months in patients receiving olaparib, compared with 4.3 months for those treated with placebo (HR 0.18; 95% CI 0.10–0.31; *P*<0.0001; Figure 1). Significant but smaller benefits in PFS were seen in patients who were *BRCA*wt who received olaparib (Table 1; Figure 1). All sensitivity analyses and centralised computed tomography (CT) scan assessments confirmed the observed increase in PFS in patients with a *BRCA* mutation receiving olaparib, compared with placebo (blinded independent central review PFS HR 0.22; 95% CI 0.12–0.40; *P*<0.0001).

There was no statistically significant difference in the interim OS analysis (58% maturity) for the overall population, or *BRCA*m or wild-type subgroups. However, there was no suggestion of an excess mortality for *BRCA*m patients treated with olaparib compared with placebo (upper one-sided 90% confidence limit for survival of 0.99 in *BRCA*m patients). It should also be noted that this study was not powered to detect an OS difference. Furthermore, the OS results may have been confounded by the use of subsequent PARPi therapies in patients who were randomised and treated on the placebo arm (23% of *BRCA*m patients receiving placebo compared with no patients receiving olaparib). A *post-hoc* analysis that explored interim OS excluding patients from all study sites where at least one patient received post-progression treatment with a PARPi, resulted in a numerical improvement in the OS HR in all groups (olaparib *vs* placebo; overall population, median OS 29.8 *vs* 26.6 months, respectively, HR 0.80; 95% CI 0.55–1.16; *P*=0.243; *BRCA*m population, median OS 34.9 *vs* 26.6 months, respectively, HR 0.52; 95% CI 0.28–0.97; *P*=0.039), suggesting that post-progression PARPi treatment could have a confounding influence on the original OS analyses; further analyses are ongoing (Matulonis *et al*, 2015). The final OS analysis will be performed after 226 deaths (85% maturity) (Ledermann *et al*, 2014b).

**Figure 2:**
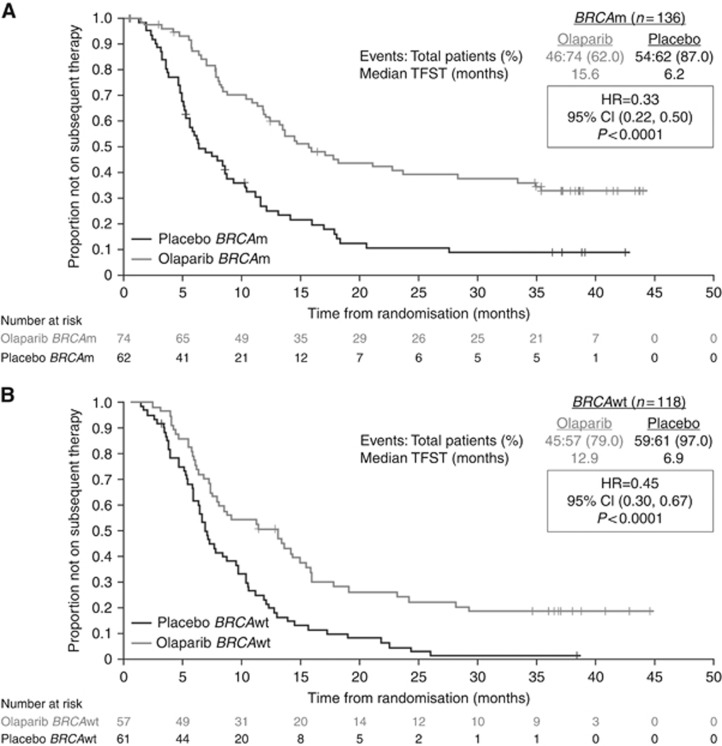
Time to first subsequent therapy or death in (**A**) patients with a *BRCA* mutation and (**B**) in patients with wild-type *BRCA* from the pivotal Phase II olaparib maintenance study in patients with platinum-sensitive relapsed serous ovarian cancer (Ledermann *et al*, 2012, 2014b). BRCAwt, wild type (includes patients with no known BRCA mutation or a mutation of unknown significance); TFST, time to first subsequent treatment or death. Reprinted from Ledermann J *et al* (© 2014 with permission from Elsevier).

**Figure 3:**
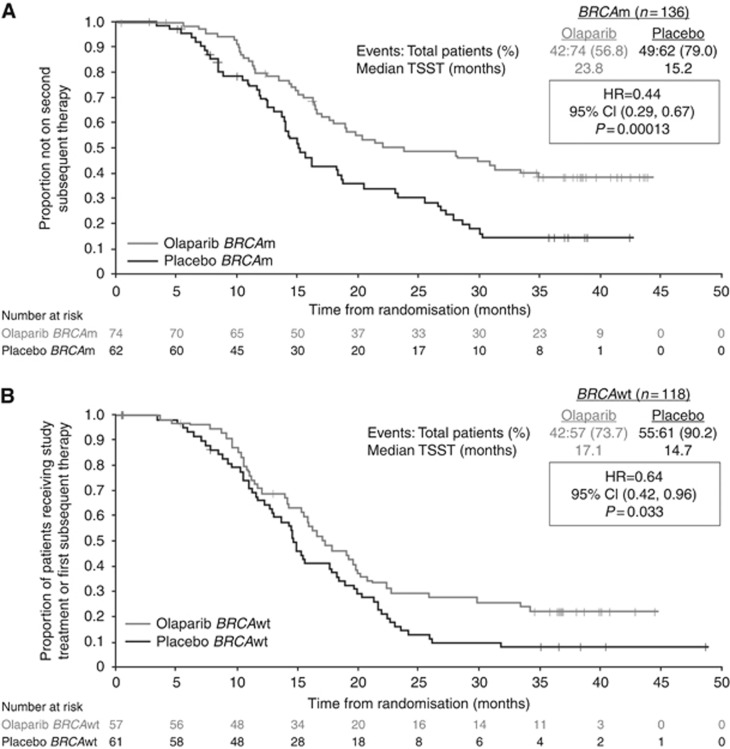
Time to second subsequent therapy or death in (**A**) patients with a *BRCA* mutation and (**B**) in patients with wild-type *BRCA* from the pivotal Phase II olaparib maintenance study in patients with platinum-sensitive relapsed serous ovarian cancer (Ledermann *et al*, 2012, 2014b). BRCAwt, wild type (includes patients with no known *BRCA* mutation or a mutation of unknown significance); TSST, time to second subsequent treatment or death. Reprinted from Ledermann J *et al* (© 2014 with permission from Elsevier).

In order to gain more clinical information beyond progression, exploratory analysis of TFST and TSST was performed in the *BRCA*m and non-*BRCA*m subgroups. In the *BRCA*m subgroup, the median TFST was 15.6 months in those who had received olaparib, and 6.2 months in those who had received placebo (HR 0.33; *P*<0.00001; Figure 2). In the *BRCA*m subgroup, the median TSST was 23.8 months, compared with 15.2 months for patients receiving olaparib and placebo, respectively (HR 0.44; *P*=0.00013; Figure 3). Thus, the PFS benefit of olaparib is maintained beyond progression and persists until the second subsequent treatment. Olaparib treatment does not compromise subsequent therapy, and the 9.4-month median difference in delay in restarting chemotherapy (TFST) in the patients treated with olaparib suggests that clinical relapse is different in the two groups of patients.

No statistically significant or clinically relevant differences in health-related quality-of-life end points were noted between treatment groups in the overall or *BRCA*m populations demonstrating that olaparib has no detrimental effect on patients’ quality of life (Ledermann *et al*, 2012, 2014b).

## Supporting studies for olaparib and other PARP inhibitors for maintenance therapy

There have been several studies that have supported the rationale, efficacy, and use of both olaparib and other PARPi as maintenance treatment in ovarian cancer. In a Phase II, multicentre, international, randomised, open-label study in patients with platinum-sensitive relapsed ovarian cancer (ClinicalTrials.gov: NCT01081951), olaparib given with paclitaxel and carboplatin chemotherapy, and continued as maintenance monotherapy, led to a significant improvement in PFS compared with chemotherapy alone (median PFS 12.2 and 9.6 months, respectively; HR 0.51; 95% CI 0.34–0.77; *P*=0.0012; Oza *et al*, 2015). In this study, approximately 25% of patients had a *BRCA* mutation and in two-thirds *BRCA* mutation status was unknown; a greater treatment benefit was seen in patients with a *BRCA*m (PFS HR 0.21; 95% CI 0.08–0.55; *P*=0.0015), compared with the overall population or patients without a *BRCA* mutation (Oza *et al*, 2015). Although this study was not designed to measure the contribution of each treatment phase (i.e., olaparib plus paclitaxel and carboplatin chemotherapy compared with paclitaxel and carboplatin chemotherapy alone, excluding the olaparib maintenance phase), the late separation of the PFS curves and improvement in objective response during the combination phase suggest that the olaparib maintenance phase was most likely the key contributor to the observed PFS improvement. Furthermore, to prevent myelosuppression during the combination phase of the study, the carboplatin dose was reduced when in the presence of olaparib (Oza *et al*, 2015). However, the combination of olaparib and platinum chemotherapy in patients with platinum resistant recurrent ovarian cancer could be investigated further in future clinical trials.

Two Phase III trials evaluating monotherapy olaparib *vs* placebo in the maintenance setting in patients with ovarian cancer have completed recruitment. The SOLO 2 study (NCT01874353) has a very similar design to Study 19, but includes only patients with a *BRCA* mutation. SOLO 1 (NCT01844986) is a maintenance study in which olaparib or placebo is given for 2 years after first-line chemotherapy in patients with a *BRCA* mutation. Both these trials use the new tablet formulation of olaparib, given at 300 mg (150 mg × 2) twice daily, rather than 400 mg (50 mg × 8) twice-daily capsules.

Two other PARPi, niraparib (MK 4827) and rucaparib, that have both been shown to have activity as a single agent (Sandhu *et al*, 2013; McNeish *et al*, 2014) are currently being evaluated in Phase III maintenance trials in platinum-sensitive ovarian cancer. The NOVA study (NCT01847274) of niraparib and the ARIEL 3 study of rucaparib include cohorts of patients with or without a *BRCA* mutation. Randomisation in ARIEL 3 is stratified by HRD status, which was established in the ARIEL 2 study, a single-arm study with rucaparib in which tumour biopsies are tested for HRD (McNeish *et al*, 2014). Other PARPi in earlier-phase development are BMN-673, veliparib (ABT888), and ABT-767.

## Regulatory approval of PARP inhibitors

There are now several PARPi in clinical development for the treatment of ovarian cancer. The earliest studies have been with the capsule formulation of olaparib, which has now been approved by the European Medicines Agency as monotherapy for the maintenance treatment of adult patients with platinum-sensitive relapsed *BRCA*m high-grade serous ovarian cancer (including fallopian tube or primary peritoneal) in response (complete response or partial) to platinum-based chemotherapy, and in the USA as monotherapy treatment for patients with advanced relapsed high-grade serous ovarian cancer with a deleterious/suspected deleterious germline *BRCA* mutation (as detected by a FDA-approved test) who have received three or more prior lines of chemotherapy treatment. The recommended dose of olaparib capsules is 400 mg taken twice daily. It has been recommended that treatment be continued until progression of the underlying ovarian cancer. Additionally, a tablet formulation of olaparib is currently in Phase III development.

## Safety and tolerability of PARPi

Olaparib is generally well tolerated in both *BRCA*m and wild-type patients. The most frequently reported adverse events (AEs) reported in the pivotal study (Ledermann *et al*, 2012, 2014b) were fatigue, low-grade nausea, vomiting, and anaemia, in both groups of patients (Table 2). Treatment to progression was achieved in most patients, with interruption of olaparib therapy or a dose reduction of olaparib used to manage AEs in 28% and 23% of patients, respectively. Discontinuation of therapy due to AE was rare; seven patients receiving olaparib and two on placebo. The long-term use of olaparib is feasible, with 18% of patients in the olaparib group remaining on treatment for greater than 3 years. This is further supported by the quality-of-life assessments that have not shown any detrimental effects during olaparib therapy (Ledermann *et al*, 2014a).

Other PARPi have a similar toxicity profile to olaparib, although thrombocytopenia is more evident with niraparib (Sandhu *et al*, 2013). However, additional AEs have been reported with PARPi that should be monitored. These include diarrhoea, constipation, anaemia, thrombocytopenia, dyspepsia, and photosensitive rash (Sandhu *et al*, 2013; Plummer *et al*, 2014a; de Jonge *et al*, 2014; Kristeleit *et al*, 2014; Plummer *et al*, 2014b). Increased alanine aminotransferase (ALT)/aspartate aminotransferase (AST) was seen in approximately 35–40% of patients receiving rucaparib in its Phase I/II study (Kristeleit *et al*, 2014). The increased ALT/AST was generally transient, not requiring treatment discontinuation.

Myelodysplastic syndromes (MDS) and acute myeloid leukaemia (AML) are potential risk factors that need to be monitored for, as they have been recorded in patients with ovarian cancer and those with a *BRCA*m; the incidence of MDS/AML is <1% of olaparib-treated patients in the whole clinical programme to date (>3000 patients). Patients who have developed MDS/AML had prior risk factors for developing these events, such as prior chemotherapy and/or radiotherapy. Further studies are required to confirm whether there is any association between MDS/AML and treatment with PARPi.

## Future treatment options with PARP inhibitors

Preclinical studies combining angiogenic inhibitors with PARPi have demonstrated an additive effect. Down-regulation of homologous recombination repair genes, such as *BRCA1* and *RAD51*, occurs with hypoxia. Enhancement of PARPi sensitivity in the hypoxic setting, combined with antiangiogenic action through vascular endothelial growth factor 3 (VEGF3) inhibition may also result in downregulation of *BRCA1* and *BRCA2* in cancer cells. This has led to clinical studies in which olaparib has been combined with an anti-angiogenic agent. The randomised, Phase II study (NCT01116648) reported by Liu *et al* (2014) evaluated the combination of olaparib with cediranib, an oral tyrosine kinase inhibitor of VEGF receptor, *vs* olaparib alone for patients with recurrent platinum-sensitive ovarian cancer. The trial demonstrated that the combination of cediranib plus olaparib improved PFS in women with recurrent platinum-sensitive high-grade serous or endometrioid ovarian cancer. The median PFS was 17.7 months (95% CI 14.7 – not reached) in the combined treatment group, compared with 9 months (95% CI 5.7–16.5) for those treated with olaparib alone (HR 0.42, 95% CI 0.23–0.76; *P*=0.005). Further trials combining cediranib and olaparib, either as treatment or maintenance following chemotherapy, are being planned following the results of this trial. A first-line trial is being planned to investigate the addition of olaparib during the maintenance phase of bevacizumab therapy following chemotherapy and bevacizumab (NCT02121990). Other combinations with olaparib are planned, including a Phase I study in combination with the phosphoinositide 3 kinase inhibitor, buparlisib (NCT01623349) in high-grade serous ovarian cancer, or triple-negative breast cancer (Matulonis *et al*, 2015). The combination with olaparib and the alkylating agent carboplatin (NCT01445418) is also being studied (Lee *et al*, 2014).

## Conclusions

The key efficacy data from the pivotal olaparib studies in patients with platinum-sensitive recurrent high-grade serous ovarian cancer described here demonstrate clinical efficacy of olaparib with improvements in the primary endpoint PFS, and supporting evidence of clinical benefit from the exploratory end points that include TFST and TSST. The AEs have been discussed with particular emphasis on monitoring and treating nausea, vomiting, fatigue, and anaemia, with very few patients stopping treatment because of AEs. The dose is not based on weight or surface area, so dose interruptions and dose reductions may be needed in some patients, therefore allowing chronic dosing. Based on the evidence presented, patients who will receive the greatest benefit from PARP inhibition are those with platinum-sensitive relapsed high-grade serous ovarian cancer and a *BRCA* mutation. The proportion of patients with non-*BRCA1*/*2* homologous recombination repair gene mutations in Study 19 was relatively small (*BRIP1* (*n*=5), *RAD54L* (*n*=3), *CDK12* (*n*=3), *RAD51B* (*n*=2 pts); Dougherty *et al*, 2014). Such genes deserve further study for their potential role in prolonged treatment benefit and survival for patients receiving platinum therapy or PARPi.

## Figures and Tables

**Table 1:** Summary of key efficacy outcomes from the pivotal Phase II olaparib maintenance study in patients with platinum-sensitive relapsed serous ovarian cancer

	**Overall population (** ***n*** **=265)**		***BRCA*** **-mutated subgroup (** ***n*** **=136)**		***BRCA*** **wild-type/VUS subgroup (*****n*****=118)**	
	**Olaparib**	**Placebo**	**Olaparib**	**Placebo**	**Olaparib**	**Placebo**
**Progression-free survival (PFS)**						
Events/total patients, *n* (%)	60/136 (44%)	94/129 (73%)	26/74 (35%)	46/62 (74%)	32/57 (56%)	44/61 (72%)
Median PFS (months)	8.4	4.8	11.2	4.3	7.4	5.5
HR (95% CI)	0.35 (0.25–0.49)		0.18 (0.10–0.31)		0.54 (0.34–0.85)	
*P* value	<0.0001		<0.0001		0.0075	
**Overall survival (OS; interim** (**58%**) **maturity)**						
Events/total patients, *n* (%)	77/136 (57%)	77/129 (60%)	37/74 (50%)	34/62 (55%)	36/57 (63%)	41/61 (67%)
Median PFS (months)	29.8	27.8	34.9	31.9	24.5	26.2
HR (95% CI)	0.88 (0.64–1.21)		0.73 (0.45–1.17)		0.99 (0.63–1.55)	
*P* value	0.44		0.19		0.96	

Abbreviations: CI=confidence interval; HR=hazard ratio; VUS=variant of unknown significance. From Ledermann *et al* (2014b).

**Table 2:** Summary of adverse events (all CTCAE grades) reported in ⩾20% of patients in the pivotal Phase II olaparib maintenance study in patients with platinum-sensitive relapsed serous ovarian cancer

	**Olaparib (** ***n*** **=136)**		**Placebo (** ***n*** **=128)**	
**Adverse event (AE),** ***n*** **(%)**	**All grades (%)**	**Grade ⩾3**	**All grades (%)**	**Grade ⩾3**
Patients with any AE	132 (97)	55 (40)	119 (93)	28 (22)
Nausea	96 (71)	3 (2)	46 (36)	0
Fatigue	71 (52)	10 (7)	50 (39)	4 (3)
Vomiting	46 (34)	3 (2)	18 (14)	1 (<1)
Diarrhoea	37 (27)	3 (2)	31 (24)	3 (2)
Abdominal pain	34 (25)	3 (2)	34 (27)	4 (3)
Anaemia	29 (21)	7 (5)	7 (5)	1 (<1)
Headache	28 (21)	0	16 (13)	1 (<1)
Constipation	28 (21)	0	14 (11)	0
Decreased appetite	28 (21)	0	17 (13)	0

Abbreviation: CTCAE=Common Terminology Criteria for Adverse Events. From Ledermann *et al* (2014b).
